# Essential nonclinical competencies for cardiovascular specialists *imperatives for training*

**DOI:** 10.1002/clc.23914

**Published:** 2022-09-07

**Authors:** C. Michael Valentine, Richard A. Chazal

**Affiliations:** ^1^ Division of Cardiology University of Virginia School of Medicine Charlottesville Virginia USA; ^2^ Heart and Vascular Institute, Lee Health Fort Myers Florida USA

## Abstract

C. Richard (Dick) Conti was a pioneer in innovation…not only in clinical and academic fields, but also in the exposure of academicians, clinicians, and trainees to various environments for expansion of their knowledge base…and world view. In an evolving environment of systems of medical care, engagement in management and planning by physicians and all members of the care team is essential to ensure quality for patients and to develop processes that work effectively for practitioners. This is particularly true in cardiovascular disease, where the majority of physicians are now part of integrated healthcare systems. Such integration can have advantages, but can also lead to a perceived and real loss of professional control over the practice of medicine. As health systems grow, even those practitioners who remain “independent” require the ability to actively engage in system programs, processes, and planning. Tools to effectively contribute to such skill sets are not commonly part of formal training. This communication describes the needs for training in nonclinical competencies, some current resources, and a model for formal integration of such instruction into cardiology fellowship training. An approach such as this honors the memory of Dick Conti, as an educator and leader who continuously looked for avenues to improve the practice of cardiovascular medicine.

## 1 INTRODUCTION

The traditional training of cardiovascular specialists has focused on clinical and procedural skills needed for the direct care of patients.^1^ Areas of required competency are outlined in detail in the ACC's Core Cardiovascular Training Statements (COCATS).^2^ While the most recent iteration of COCATS includes guidance for needed learning in important nonclinical areas of cardiovascular medicine, gaps in training persist. There are multiple reasons for such training and learning gaps. Most importantly, resource allocation for nonclinical learning is often limited, competing with needs for direct patient care instruction. The time available for instruction and learning is a significant factor, particularly in an era where restriction in hours spent by trainees is enforced for reasons related to safety.^3^ Finally, while some faculty are personally comfortable in the realm of nonclinical competence, most received their knowledge by “on the job training” or mentorship, never having had the advantage of formal training to develop expertise in these areas. Therefore, they have limited models for instructing in these nonclinical areas.

During his storied career, Dr. Conti often demonstrated innovation and “out of the box” thinking to benefit his institutions, colleagues, mentees, and patients. Upon arriving in Gainesville, Florida, in 1974, Conti became the Chief of Cardiology at a relatively small and new program. Over the years, he recruited extraordinary talent, including his eventual successors as Chief, Carl Pepine, and his daughter Jamie Conti. Dick Conti built a powerful cardiovascular institution, well recognized today. He contributed significantly to the American College of Cardiology (ACC), serving in multiple leadership roles, including President of the College in 1989. He was instrumental in developing international ties between American cardiologists (who were somewhat insular at the time) and the international academic and clinical community. His work in China was particularly groundbreaking. In the 1990s Conti fostered a program for engaging junior faculty and “rising stars” from the United States to travel, learn, and lecture overseas. This endeavor fostered long‐term relationships that continue to bear fruit today. One of those young cardiologists benefiting from the program was Pamela Douglas, MD MACC, who went on to serve as Chief of Cardiology at two prominent institutions, eventually becoming the second female president of ACC and currently sitting as the Ursula Geller Professor of Research in Cardiovascular Diseases in the Department of Medicine at Duke University. Of those experiences with Dr. Conti, Dr. Douglas relates “Thirty years ago, travel by American cardiologists, particularly junior physicians, overseas was unusual. The exposure to different healthcare models and academic institutions helped broaden my perspective and influence my ability to improve our own systems." R. A. Chazal (personal communication, August 13, 2022).

As an early supporter (along with fellow pioneer Pepine) of state chapters in the ACC, Conti promoted grassroots engagement of clinicians with organized and academic medicine. He continued that support in a very personal way, continuing to attend (*and very actively participate in*) ACC Florida Chapter meetings throughout his life. His editorship of ACCEL (the ACC audio journal) further cemented his place in using emerging technology to further education and professional engagement.

These are but a few of many examples of Dick Conti's drive to explore new and better ways to improve medical care, particularly in the field of cardiovascular disease. His lifelong efforts to find new solutions provide valuable inspiration for those whose career is focused on the well‐being of patients.

## 2 NONCLINICAL AREAS OF IMPORTANCE

The personal tools needed to navigate organizations (from small practices to large healthcare systems and institutions) are often complementary to clinical medicine but some skills are distinct from those needed for bedside care. Efficient clinical care mandates good use of many such tools, but the importance of these skills beyond the bedside drives much of the need for nonclinical competencies.

The importance of input from active clinicians, researchers, and academicians into the “running” of operations cannot be overstated. Abrogation of complete managerial responsibility to nonphysicians/nonclinicians places burdens on those often unfamiliar with nuances related to patient care. All are concerned about the risks of prioritizing finance over quality, while cognizant of the importance of fiscal integrity (“no margin, no mission”).^4^ Engagement by clinicians helps ensure balance in the planning and management of hospitals and healthcare institutions by inserting those who see patients and thus have a direct link.^5^, ^6^

Among the many nonclinical skills useful (and often essential) for meaningful engagement at a system level are *organization* (including skills related to meetings), *digital* skills, *project management*, *mentoring*, and *interpersonal* relationship training. While not a comprehensive list, these are skill sets that are needed, but not universally intrinsic to professional medical education. Some, such as project management, may be part of the training of some individuals (such as researchers), but not for others (task‐based clinicians). This noncomprehensive list is intended to provide insight into need; the actual process changes are beyond the scope of this communication.

*Organizational skills* relate to abilities in team building and the effective use of resources (including individuals). The ability to recruit and inspire participation by others is at the center of successful team‐based enterprises. Effective scheduling and time management, particularly in (sometimes endless) meetings, promote ongoing engagement by busy professionals. Meetings must be at a convenient time and location, must start *and end* on time, and must have a defined purpose that is demonstrably accomplished. Such organization encourages ongoing participation by busy physicians, advanced practice providers, and nurses. Beyond the usual challenges in such gatherings, the recent COVID pandemic has altered ways in which we interact. Organizing and running virtual meetings now present entirely new obstacles compared to in‐person assemblies.

*Digital skills* have taken on an increasingly important role in society as a whole and in medicine in particular. Young clinicians are often thrust into an environment dominated by electronic records and find themselves devoid of personal tools needed for efficient use. Communication, once verbal and handwritten, has largely evolved (for better and worse) to text and email messages, whose composition and script is distinct from traditional letters. Efficient and effective communications are essential. The use of remote/virtual communication in an effective manner is a new and evolving area of expertise. Transmission of knowledge by lecture, including tools such as PowerPoint, have highly variable effectiveness, based on proficiency. Editing of documents, using tools such as Track Changes alters the efficiency and accuracy of work. The effective use of hand‐held devices and links to digital media (such as guidelines) is a competency that many young trainees already possess, but with an opportunity for focused refinement.

*Project management*, an intrinsic part of life for some medical professionals such as researchers, can be elusive to clinicians whose daily work is focused on a series of finite tasks (do a procedure on Mrs. X, read an echo on Mr. Y, see the scheduled clinic patients). The ability to plan, execute and sustain an endeavor whose timeline can be days, weeks, or years (instead of minutes or hours) is challenging to those not accustomed to or schooled in methods for success long term. Many pure clinicians shied away from academic careers because they consciously or subconsciously were better suited to a series of short‐term tasks (despite the volume of such), rather than the long‐term management of projects. Effective participation in planning and management of healthcare institutions, small and large, benefits from the ability to manage long‐term endeavors (ie a project to improve MI care). Specific training in how to shift from a task‐oriented mentality to a project mentality can increase the likelihood of success in protracted ventures.

*Mentoring* others in the pursuit of improved team care, promotion of colleagues, diversification of workforce, and succession planning is crucial to institutional success. While modeling of ideal behavior is likely (and, hopefully, universal) in cardiovascular medicine, *active* mentoring involves behaviors and actions that are distinct. Those professionals in research and teaching exercise this on a daily basis with colleagues, junior collaborators, and trainees. Physicians and team members who are nearly exclusively involved in clinical practice may not have the refinement of skills needed to promote the advancement of others. These learned behaviors benefit systems and mentees…and are highly beneficial to the mentor.

Expertise in managing *interpersonal relationships* is important for everyone in society. This becomes even more important when meaningful engagement and leadership in healthcare systems occurs. The ability to identify personnel problems and to subsequently effectively, fairly, and kindly deal with such is a desired (and often elusive) proficiency that benefits from expert training…as well as continuous experience and learning. Of all of the nonclinical skills here listed, this is perhaps the most challenging…and the skill most valuable professionally and personally.

## 3 CURRENT SOURCES OF NONCLINICAL EDUCATION

Increasing awareness of the gaps in nonclinical education led organizations like the ACC and the American Heart Association (AHA) to form *conferences* in the late 1980s and 1990s designed to enhance knowledge through widespread publication of the results of these collaboratives. Drs. Dick Conti, Carl Pepine, and many others advocated for these joint conferences, which promoted science and professionalism. These efforts are ongoing. In 2020, a Consensus Conference was convened at a critical time in the midst of the ongoing COVID 19 pandemic. Five separate joint task forces were formed and summary recommendations were published in *The Journal of the American College of Cardiology (JACC)* and in *Circulation* in May 2021.^7^ The task forces tackled difficult topics not easily taught on a clinical service. The list of topics is extensive and includes: conflicts of interest in teaching; publications and research data; diversity, equity, inclusion and belonging with elimination of bias; enhancing the well‐being of clinicians and the “Quadruple Aim”; patient autonomy, privacy, and social justice in health care; value‐based healthcare, patient‐centered care and “pay for performance”models, quality outcomes and the reduction of unnecessary testing and procedures; proper management of electronic health records and their impact on patient care and clinician well‐being. This type of deliberation and education is an important example of collaborative thinking and education in our complex systems, to teach fellow professionals and trainees.

Other opportunities for nonclinical Education developed at ACC in the early 2000s. Drs. Patrick 0'Gara and Rick Nishimura developed an *Emerging Faculty Program* devoted to teaching skills essential to young academicians, including successful grant applications, academic advancement, publications, time management, as well as public speaking and virtual presentation enhancement. This remains a highly sought‐after program, with foundational support insuring longevity. *Leadership Forums* developed at ACC, AHA, and most subspecialty organizations have been developed to guide young cardiovascular leaders in the areas most crucial for personal and system success, including individual leadership style and team dynamics, communication skills, conflict resolution, implicit bias, and change management skills. Some forward‐thinking medical and professional schools have adopted many of these training topics before clinicians reach clinical rotations, to help prepare them for inevitable conflicts.

*Leadership academies* are currently available through many professional societies, including ACC, The Society for Cardiac Angiography and Intervention (SCAI), Heart Rhythm Society (HRS), and others. These academies encourage young leaders to develop the nonclinical skills essential for individual, team, and system successes. The gaps identified by these societies contributed to the nonclinical competencies embedded in the *2016 ACC* COCATS document.^2^ The final section of COCATS lists those competencies expected of all clinical cardiologists and selected cardiologists based on practice focus. Unfortunately, most training programs lack the resources for in‐depth training in many of the nonclinical areas, particularly in conflict resolution, strategic planning, effective performance management, population health, financial/legal and regulatory policies, appropriate use criteria and performance measures, meeting facilitation, and professional well‐being. Societal Leadership Academies help to fill the gap, allowing young leaders the time and training to gain these skills and learn how to use them effectively.

In 2008, the ACC developed the *Cardiovascular Summit*, a meeting centered around sharing best practices in nonclinical competencies. This particular meeting has grown annually, with over 600 attending in‐person in 2019 and even more via virtual attendance in the pandemic years. The four major areas of concentration for “The Summit” were developed through surveys…"needs assessment". These include the business and financial issues of cardiovascular systems and care, operational excellence, quality improvement‐including efficiencies of care in practice, individual and dyad leadership development, and workforce well‐being. An academic track, focused on specific needs of team members in such centers, has been quite successful. An innovative poster session allows sharing of new ideas from across the country for organizational success, much as poster presentations at scientific sessions serve clinical learning.

Partners in the Summit have included *Medaxiom* and the *American Association of Physician Leadership*—two companies with a track record of excellence in training, consultation, and data management. Both organizations provide independent avenues for pursuing learning excellence.

The Summit meeting encouraged ACC to create new media efforts to reach the younger generation of aspiring leaders, prompting the development of the podcast series *Practice Made Perfect* in 2017. Over 50 podcasts have been created to date with topics ranging from practice finance, contracts, negotiation skills, understanding medical liability, clinician well‐being, time management, leading healthcare teams, and effective patterns for advanced practice providers.

Outside of professional cardiac societies, opportunities exist for broad learning in the nonclinical areas. *Degrees* including Masters in Business Administration (MBA) and Masters in Healthcare Administration (MHA), among others, can provide valuable formal education. For those focusing on a career in administration, these can be ideal, while the active clinician, academician, or researcher may not find this approach suitable.

## 4 SUCCESFUL PROGRAM MODELS

As successful as each of these efforts has been, academic training programs often still lack available resources and expertise to develop effective curricula for these nonclinical competences. As a member of the ACC Leadership Academy Cohort 111, Garima Sharma, MD, FACC, used her Capstone project to develop a curriculum for the *Johns Hopkins* Cardiovascular Fellowship Program. She helped develop longitudinal workshops on building resilience, emotional intelligence, leadership style and conflict resolution, grant writing skills, personal finance, “building your own brand” for long‐term success, understanding billing and coding and improving “life skills” of graduating fellows. She has presented her curriculum to program training directors and at the *CV Summit* in the academic track.

After joining the faculty at the *University of Virginia* in 2020, we helped develop a series of programs designed to enhance the nonclinical skills of trainees and faculty members. After surveying fellows, we have now developed 10 nonclinical sessions that are imbedded in the fellows' weekly lunch conferences. Popular topics include job search and negotiation skills, electronic health record optimization, billing and coding, conflict resolution (including “difficult conversations”), medical liability, time management, and personal finance. We have arranged mentoring sessions to aide in application enhancement, interviewing skills, and creating national contacts for job search and career advancement. The nonclinical lectures have now been extended to workshops for third‐year medical residents as well. For faculty members, an *Early Career Leadership Academy* was developed with 11 team members completing a year‐long program with new individual and team management skills, public speaking and virtual presentation training, and creation of successful capstone projects guided by partners and mentors. The Mid‐Career Leadership Academy begins Fall 2022, and will be complemented by individual executive coaching and faculty from the Batten School of Leadership and Darden School of Business at the University of Virginia. Each project was supported by a gift from the Ivy Foundation. Time will tell how much each of these benefits the individuals and the division. We believe the investment will bring substantial rewards.

## 5 DISCUSSION

This is an era when physicians and team members risk becoming commodities rather than professionals.^8^, ^9^, ^10^ Active and *effective* engagement in healthcare systems at the practice, hospital, system, institutional and national level is a vehicle for helping to ensure that the quality of care for patients supersedes pure financial concerns. Such engagement also empowers clinicians and academicians, restoring a sense of professional control. While clinical skills are integral to formalized training and lifelong learning/continuing education, opportunities for nonclinical skill development lag. Several institutions have ongoing programs to help bridge this gap. Integration of nonclinical teaching into formal academic training and lifelong learning can help provide cardiovascular professionals with tools to positively influence the future of care.

Dick Conti left a legacy of devising and implementing actions toward the ultimate goal of improved care for patients. We, as cardiovascular specialists, have the opportunity and the responsibility to perpetuate his mission.

## References

[1] Wann S . Consolidation and hybridization in the health care enterprise: how are cardiologists affected? Cardiology Today . 2018.

[2] Williams ES , Halperin JL , Arrighi JA , et al. 2016 ACC lifelong learning competencies for general cardiologists: a report of the ACC competency management committee. J Am Coll Cardiol. 2016;67:2656‐2695. 26902874 10.1016/j.jacc.2016.02.011

[3] New duty hour limits: discussion and justification. Accessed August 14, 2022. https://www.acgme.org/globalassets/pdfs/jgme-11-00-29-37.pdf

[4] No margin, no mission: flying nuns and sister Irene Kraus. 2012. Accessed August 13, 2022. https://www.teletracking.com/resources/no-margin-no-mission-flying-nuns-and-sister-irene-kraus

[5] Baldwin KS , Dimunation N , Alexander J . Health care leadership and the dyad model. Physician Exec. 2011;37:66‐70. 21827104

[6] Chazal R , Montgomery M . The dyad model and value‐based care. J Am Coll Cardiol. 2017;69:1353‐1354. 28279298 10.1016/j.jacc.2017.02.007

[7] Benjamin IJ , Valentine CM , Oetgen WJ , et al. 2020 American Heart Association and American College of Cardiology Consensus Conference on Professionalism and Ethics: a consensus conference report. Circulation. 2021;143:e1035‐e1087. 33974449 10.1161/CIR.0000000000000963

[8] Sullivan GM , Berger JS , Yarris LM , Artino AR Jr , Simpson D . Are physicians commodities? The Perspective of a Group of JGME Editors. J Grad Med Educ. 2018;10:374‐375. 30154962 10.4300/JGME-D-18-00501.1 PMC6108374

[9] It's not just primary care physicians that are in demand, specialists are quickly becoming a commodity. 2021. Accessed August 14, 2022. https://www.healthcarefinancenews.com/news/its-not-just-primary-care-physicians-are-demand-specialists-are-quickly-becoming-commodity

[10] Accessed August 14, 2022. https://www.axios.com/2021/11/24/doctors-are-becoming-a-hot-commodity

